# Comparison of Clinical Outcomes of Laparoscopic Totally Extraperitoneal (TEP) and Transabdominal Preperitoneal (TAPP) Techniques in Bilateral Inguinal Hernia Repair: A Retrospective Study

**DOI:** 10.7759/cureus.69134

**Published:** 2024-09-10

**Authors:** Yahya Ozel, Yalcin Burak Kara

**Affiliations:** 1 Department of General Surgery, Dogus University School of Medicine, Istanbul, TUR; 2 Department of General Surgery, Bahcesehir University School of Medicine, Istanbul, TUR; 3 Department of General Surgery, VM Medical Park Pendik Hospital, Istanbul, TUR

**Keywords:** bilateral inguinal hernia, intraoperative complications, laparoscopic hernia repair, operative time, postoperative complications, surgical outcomes, tapp, tep

## Abstract

Objective: This study compared the clinical outcomes of two commonly used laparoscopic techniques, transabdominal preperitoneal (TAPP) and totally extraperitoneal (TEP) repair, in the treatment of bilateral inguinal hernias.

Materials and methods: This retrospective cohort study included 250 patients who underwent laparoscopic bilateral inguinal hernia repair using either the TEP or TAPP technique between May 2009 and May 2024. The patients were divided into two groups: 50 patients in the TEP group and 200 in the TAPP group. Data were collected from patient records, including demographics, type of hernia, surgical details, intraoperative and postoperative complications, conversion rates, and early hernia recurrence. Statistical analysis was performed to compare outcomes between the two groups.

Results: Among the 250 patients included in the study, the mean age was 51.62 ± 8.79 years, and 94% (n=235) were male. The mean operative time was significantly longer in the TEP group (93.2 ± 13.0 minutes) than in the TAPP group (57.95 ± 7.5 minutes) (*p <.*001). The mean hospital stay was also longer in the TEP group (1.36 ± 0.48 days) compared to the TAPP group (1.07 ± 0.25 days) (*p <.*001). The TEP group had a higher rate of conversion to open surgery 18%(n=9) and conversion to TAPP 10% (5) than the TAPP group, which had no conversions (*p <.*0001). Postoperative complications were more frequent in the TEP group, with urinary retention being significantly higher at 16%(n=8) than in the TAPP group at 2% (n=4) (*p <.*0004). Additionally, the TAPP group experienced two (1%) notable intraoperative complications that required reoperation: arterial injury and small bowel injury.

Conclusion: The findings suggest that, while both TEP and TAPP effectively repair bilateral inguinal hernia, TAPP is associated with shorter operative times, shorter hospital stays, and fewer postoperative complications. However, the TAPP technique also presented notable intraoperative risks, including arterial and bowel injury. The choice between TEP and TAPP should be based on the surgeon’s experience, patient characteristics, and the specific clinical context.

## Introduction

Inguinal hernia repair is one of the more commonly performed operations in general surgery [1]. Bilateral inguinal hernia repair constitutes a significant portion of inguinal hernia surgeries, with the incidence of bilateral cases reaching up to 30% [2].

Selecting the most appropriate technique for groin hernia repair is a significant challenge. The ideal surgical method should be relatively easy to learn, cost-effective, yield reproducible results, and have rapid recovery and a low risk of complications (including pain and recurrence). The choice of technique is influenced by various factors, such as hernia characteristics, type of anesthesia, available resources, and the surgeon’s preference, training, and skills. Additionally, the patient’s preferences must be taken into account. Emotions can play a role in the decision-making process as can cultural differences between surgeons, countries, and regions [3,4].

The latest international guidelines for inguinal hernia management from the HerniaSurge Group recommend a mesh-based technique as the first choice for all groin hernias and the laparo-endoscopic approach for treating bilateral inguinal hernias [5]. The 2020 consensus international guidelines for the management of groin hernias also recommend laparo-endoscopic repair for primary bilateral inguinal hernias [6]. The most common laparoscopic techniques for inguinal hernia repair are the totally extraperitoneal (TEP) and transabdominal preperitoneal (TAPP) approaches [7].

The laparoscopic approach offers the advantage of addressing both groins through the same incisions used for unilateral hernia repair. Additionally, it is considered a cost-effective option compared to open repair for bilateral inguinal hernias [8].

The TAPP technique requires access to the peritoneum, whereas the TEP approach is entirely independent of the peritoneum and performed without entering the peritoneal cavity. Both methods are performed under general anesthesia and involve the use of synthetic mesh. TAPP may be more advantageous for larger hernias, whereas TEP demands more technical skill [9].

The laparo-endoscopic management of potential bilateral hernias, particularly regarding occult hernias, is a subject of ongoing discussion. While the contralateral side can be inspected during a TAPP procedure, this is typically not done during a unilateral TEP repair.

In recent decades, numerous studies comparing the two laparoscopic approaches have yielded conflicting results. Most earlier randomized trials and meta-analyses did not find significant differences in outcomes, such as total complications, time to return to work, and recurrence rate [10]. In contrast, a recent meta-analysis found less postoperative pain and shorter hospital stays with TEP repair, whereas TAPP repair was associated with a shorter duration of surgery [11]. These findings were derived from studies on unilateral inguinal hernias, however, and only a few extant studies compare the two procedures in the context of bilateral inguinal hernia.

This study evaluated the outcomes of TEP and TAPP in bilateral inguinal hernia repair in our hospital and compared intraoperative complications, operative time, hospital stay, intra- and postoperative complications, occult contralateral hernias, and early hernia recurrence.

## Materials and methods

Study design and setting

This retrospective cohort study included patients who underwent laparoscopic bilateral inguinal hernia repair in our hospitals using either the TEP or TAPP technique between May 2009 and May 2024.

Patient selection

The patients included in this study had undergone bilateral laparoscopic inguinal hernia repair by one of two surgeons during the specified period. The inclusion criteria embraced adult patients aged 18 years and above who had complete medical records and had undergone bilateral inguinal hernia repair with either the TEP or TAPP technique. Patients were excluded who had unilateral primary or recurrent hernias, used anticoagulant medication, underwent other major surgeries simultaneously, had a history of midline inferior laparotomy, or lacked follow-up. All patients were followed up for one month postoperatively to assess hernia recurrence.

In the study period, 278 laparoscopic bilateral inguinal hernia repairs were performed. Nine cases with previous inguinal hernia repairs were excluded as were five patients who had a prior midline laparotomy for prostate surgery, colon resection, or perforated appendectomy. Eight patients were excluded for lack of follow-up, and two patients were excluded for concomitant surgeries with urology. Lastly, four cases using anticoagulants were excluded. The 250 patients ultimately included in the study comprised 200 patients with TAPP and 50 with TEP.

Data collection

The patients’ data were collected from both physical patient files and the electronic medical record system. The information collected included patient demographics such as age, sex, body mass index (BMI), American Society of Anesthesiologists (ASA) classification, type of hernia (direct, indirect, or combined), surgical details (operative time, technique used), intraoperative and postoperative complications, conversion to open surgery, length of hospital stay, and follow-up outcomes (with a particular focus on early recurrence within the one-month follow-up period). Due to the study’s retrospective nature, informed consent was not obtained.

Surgical techniques

The TEP and TAPP repairs were performed according to standard protocols established at the hospital. A routine Foley catheter was not used for the patient. Except for using a 15 × 15 cm polypropylene mesh and closing the peritoneum with tacks, the TAPP surgical procedure adhered to the widely accepted standard (three-trocar laparoscopic) technique. The surgical technique followed that described in previous studies [7,12-14].

The TEP surgeries deviated from the standard surgical technique by not using tissue adhesive or a balloon for peritoneal dissection, and a 15 × 15 cm polypropylene mesh was used. The camera was used as a dissector. Otherwise, the procedure was performed according to the widely accepted standard (three-trocar laparoscopic) technique. The surgical technique followed that described in previous studies [7,12,14].

Statistical analysis

Statistical analysis was performed using Python with the assistance of an AI model and utilized the Pandas, NumPy, SciPy, and Statsmodels libraries. Continuous variables were expressed as means ± standard deviations and compared using Student’s t-test or the Mann-Whitney U test depending on data distribution. Categorical variables were expressed as frequencies and percentages and compared using the chi-squared test or Fisher’s exact test. A p-value of <.05 was considered statistically significant.

## Results

A total of 250 patients who underwent laparoscopic bilateral inguinal hernia repair were included in the study, with 50 (20%) undergoing TEP and 200 (80%) undergoing TAPP procedures. The patients’ mean age was 51.62± 8.79 years, with 235 (94%) being male and 15 (6%) females. The mean BMI was 24.72± 2.17 kg/m². Among the patients, 117 (46.8%) were classified as ASA I, 117 (46.8%) as ASA II, and 16 (6.4%) as ASA III. The mean duration of surgery was 65± 16.67 minutes, and the average length of hospital stay was 1.12± 0.33 days (see Table 1).

**Table 1:** Characteristics of patients with laparoscopic bilateral inguinal hernia repair m Mann-Whitney u test; X² chi-squared test; t t-test; SD: standard deviation; BMI: body mass index; ASA: American Society of Anesthesiologists classification; Test st. Test statistic; TEP: totally extraperitoneal; TAPP: transabdominal preperitoneal The p-value considered significant was p<0.05

	Total	TEP	TAPP	p-value	Test st.
Patient count n (%)	250	50 (20)	200 (80)		
Age, mean ± SD	51.62 ± 8.79	52.56 ± 10.83	51.38 ± 8.22	0.35 ^m^	5431.5
Sex, n (%)					
Male	235 (94)	47 (94)	188 (94)	1.0 ^χ²^	0.000
Female	15 (6)	3 (6)	12 (6)	1.0 ^χ²^	0.000
ASA, n (%)				0.5 ^χ²^	1.379
ASA I	117 (46.8)	23 (46)	94 (47)	1.0^ χ²^	0.000
ASA II	117 (46.8)	22 (44)	95 (47.5)	0.861^χ²^	0.030
ASA III	16 (6.4)	5 (10)	11 (5.5)	0.691^χ²^	0.158
BMI, mean ± SD	24.72 ± 2.17	24.85 ± 2.1	24.69 ± 2.19	0.64 ^t^	0.470
Operative time (min), mean ± SD	65 ± 16.67	93.2± 13.04	57.95± 7.49	<0 .001^m^	9913.5
Length of stay (days), mean ± SD	1.12 ± 0.33	1.36 ± 0.48	1.07 ± 0.25	< 0.001^m^	6475.0

**Figure 1:**
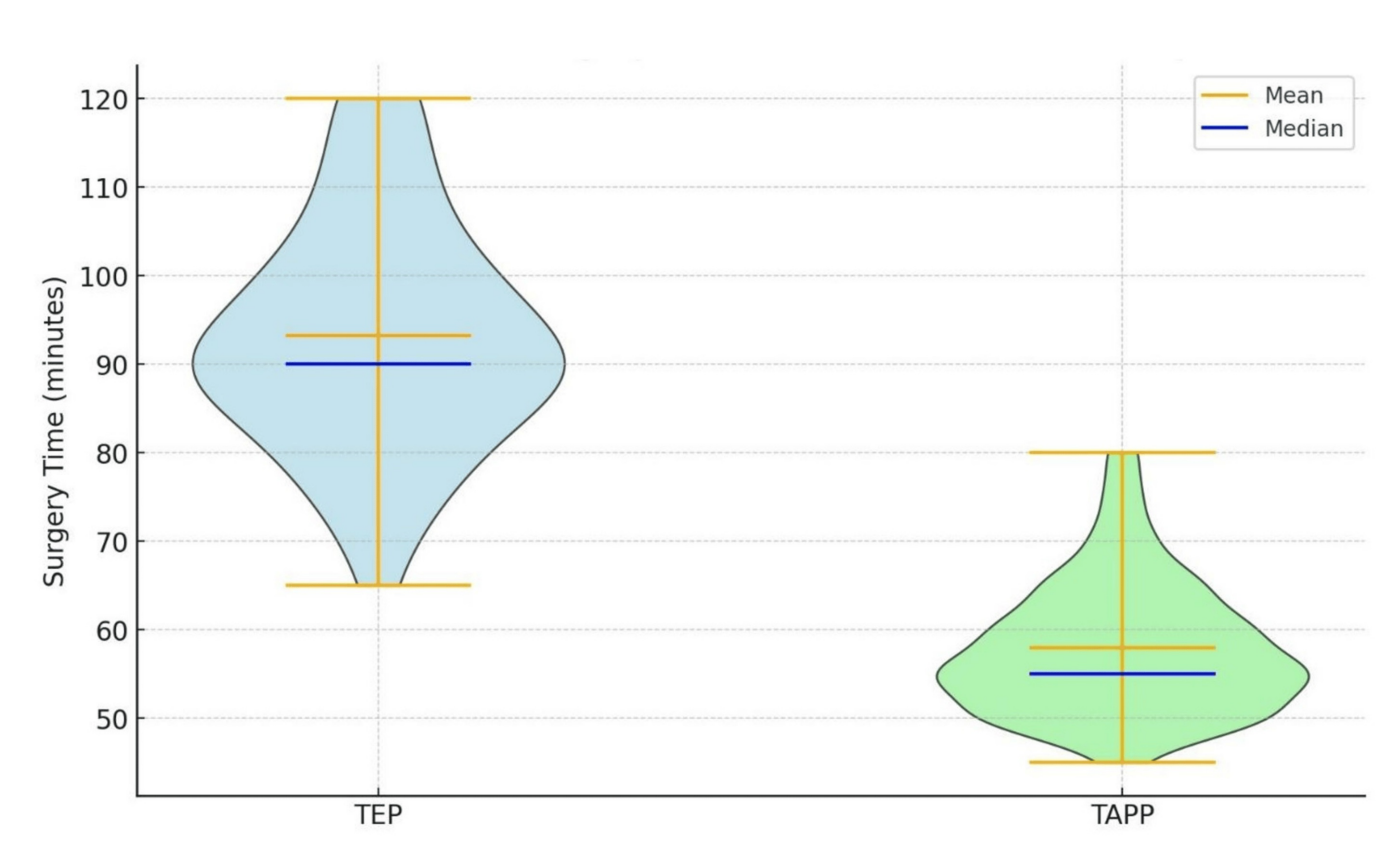
Violin plot of surgery times for TEP and TAPP groups with mean and median time TEP: Totally extraperitoneal; TAPP: transabdominal preperitoneal

**Figure 2:**
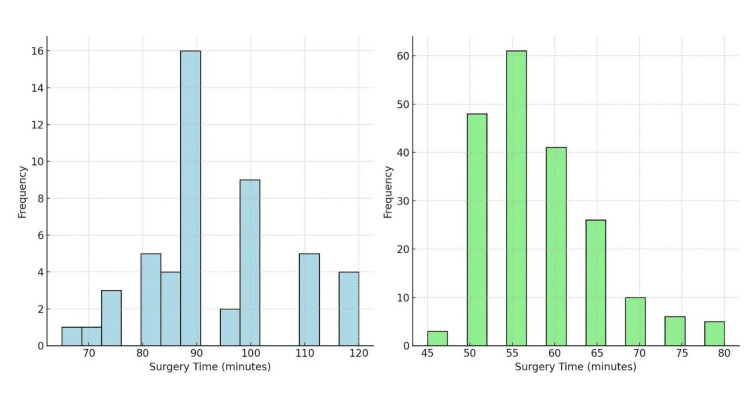
Graphic comparative histograms of TEP and TAPP TEP: Totally extraperitoneal; TAPP: transabdominal preperitoneal

The analysis found no statistically significant differences between the TEP and TAPP groups in terms of age distribution, BMI, gender distribution, or ASA classification (p >.50). However, a significant difference was observed in the duration of hospital stay, with the TEP group having a longer mean stay (1.36 ± 0.48 days) than the TAPP group (1.07 ± 0.25 days, p <.001). Additionally, the mean surgical time was significantly longer in the TEP group (93.2 ± 13.0 minutes) than in the TAPP group (57.95 ± 7.5 minutes, p <.001) (see Figures 1, 2). These findings suggest that, while the two procedures are similar in several aspects, TAPP may be associated with shorter hospital stays and operative times (Table 1).

In the TEP group, 5 of the 50 patients (10%) required conversion to open surgery due to technical difficulty, and 9 of the 50 patients (18%) were converted to the TAPP method due to peritoneal rupture. Conversely, no patients in the TAPP group required conversion to open surgery or any other method, with all 200 procedures completed as initially planned (p <.005). The transition from a unilateral to a bilateral approach occurred exclusively in the TAPP group, in which 15.5% (n=31) of cases required that change during surgery (p <.005) (Table 2).

**Table 2:** Conversions in bilateral inguinal hernia repairs Test st: Test statistic; X² chi-squared test; TEP: totally extraperitoneal; TAPP: transabdominal preperitoneal The p-value considered significant was p<0.05.

	Total	TEP	TAPP	p-value	Test st.
Patient count n (%)	250	50(20)	200(80)		
TEP to TAPP conversion, n (%)	5 (2)	5 (10)	0 (0)	<0.0001^ χ²^	32.34
Open conversion, n (%)	9 (3.6)	9 (18)	0 (0)	<0.0001^ χ²^	15.63
Unilateral to bilateral conversion, n (%)	31(12.4)	0 (0)	31(15.5)	0.0062^ χ²^	7.48

Complications

The examination of all bilateral hernia repairs revealed an overall intra- and postoperative complication rate of 12%(n=30). Intraoperative complications occurred in only two cases (0.8%), both in the TAPP group. The first case was an arterial injury to the inferior epigastric artery (IEA) when the peritoneum was opened with a hook. Hemostasis was achieved intraoperatively using a clip, and the surgery was successfully completed laparoscopically. The second case involved a small bowel injury that was not detected during the operation. On the first postoperative day, the injury was identified as requiring reoperation, and it was repaired laparoscopically with a primary suture. The patient was subsequently discharged on the fourth postoperative day.

A comparison of postoperative complications in the two groups revealed that the TEP group’s postoperative complications were statistically significantly higher than those of the TAPP group (p <.002). The TEP group exhibited a statistically significant high urinary retention, but there was no statistically significant difference between the TEP and TAPP groups in terms of seroma, pelvic hematoma, scrotal hematoma, readmission rate, or hernia recurrence in the first month (see Table 3).

**Table 3:** Outcomes of patients with laparoscopic bilateral inguinal hernia repair X² chi-squared test; Test st.: test statistic; f Fisher's exact test; TEP: totally extraperitoneal; TAPP: transabdominal preperitoneal; IEA: inferior epigastric artery The p-value considered significant was p<0.05. The chi-squared test and Fisher's exact test were used for all of the variables.

	Total	TEP	TAPP	p-value	Test st.	
Patient count n (%)	250	50(20)	200(80)			
Total complications n (%)	30 (12)	14(28)	16 (8)	0.0003	0.224	
Intraoperative complications, n (%)	2 (0.8)	0	2 (1.0)	1.0 f		
IEA injury, n (%)	1 (0.4)	0	1 (0.5)	1.0 f		
Small intestine injury, n (%)	1 (0.4)	0	1 (0.5)	1.0 f		
Postoperative complications, n (%)	28(11.6)	14 (28)	14 (7.0)	<0.001^χ²^	15.69	
Pelvic hematoma, n (%)	1 (0.4)	0	1 (0.5)	1.0^ χ²^	0.0	
Scrotal hematoma, n (%)	1 (0.4)	0	1 (0.5)	1.0^ χ²^	0.0	
Urinary retention or infection, n (%)	12 (4.8)	8 (16)	4 (2)	0.0001^χ²^	14.23	
Seroma, n (%)	14 (5.6)	6 (12)	8 (4)	0.063^χ²^	3.45	
Readmission within 30 days, n (%)	1 (0.4)	0	1 (0.5)	1.0 f		
Hernia recurrence, n (%)	1 (0.4)	1 (2)	0 (0)	0.2 f		

## Discussion

Bilateral inguinal hernias often require treatment that optimizes outcomes while minimizing recovery time and complications. Laparoscopic approaches, particularly TEP and TAPP, are increasingly favored for the treatment of bilateral inguinal hernias due to several key advantages. Both hernias can be addressed through the same set of incisions, which is less invasive and more convenient for the patient. TEP and TAPP are distinguished primarily by how they access the preperitoneal space, and this difference confers both advantages and disadvantages to each technique and plays a significant role in influencing both the intra- and postoperative outcomes of the TEP and TAPP methods [3,4]. Our study aimed to identify these differences by comparing TEP and TAPP techniques.

In our literature search, studies generally indicated that TAPP tends to result in shorter surgery durations [7,15,16]. In studies, the average surgery duration was 53-102 minutes for TEP and 67-98 minutes for TAPP in bilateral inguinal hernia repairs [17-20]. The average surgery duration for the TEP technique without the use of a balloon is reported as 120 minutes [15]. In our study, the mean surgery time for the TEP technique was 93.2 minutes, whereas the TAPP technique had a shorter duration of 57.95 minutes, findings that are consistent with the literature. The longer duration of TEP surgeries as indicated in the literature may be attributed to the complexity of the cases or the learning curve associated with the technique. The challenges of the TEP technique, along with the absence of balloon use, contributed to the extended surgery times. In our experience, using a camera to create a working area in the absence of a balloon dissector caused both time loss and prolongation of the surgery due to sharp dissection which cause small openings in the peritoneum. In contrast, using a tacker to close the peritoneum in the TAPP technique shortened the surgery duration. Besides, in our study, the number of TAPP patients was higher than the number of TEP patients which can be explained by the fact that TAPP positively affected the learning effect and caused a shortening of operation time. These findings suggest that while TAPP offers shorter operative times, TEP remains a viable option depending on the surgeon’s experience and the clinical situation.

The literature reports conversion rates from TEP to TAPP in the range of 5%-10%. It depends on the complexity of the case, the experience of the surgeon, the use of sharp instruments, and previous adhesions that have been suggested as contributing to the occurrence of these tears [5,21-23]. In some cases, small openings can occur, but the procedure can still be continued. In our study, technical difficulty and peritoneal rupture required conversion to open surgery in 5 cases (10%) and conversion to TAPP in nine cases (18%) for a total of 14 conversions (28%). This very high conversion rate may be due to the learning curve surgery and the absence of balloon use in TEP.

In our study, the mean hospital stay for TEP was 1.36 days, whereas it was 1.065 days for TAPP. These findings align well with the existing literature, which generally indicates that TAPP may yield slightly shorter hospital stays than TEP [15,17]. While some studies, found no significant difference in hospital stay between the two techniques, other factors, including return to work and overall recovery, may differ [24].

Contralateral hernia

Using the TAPP technique for inguinal hernia repair, all defects of the myopectineal orifice, including incidental contralateral hernias, can be evaluated and covered with mesh without the need for additional skin incisions. It has previously been shown that patients with a symptomatic inguinal hernia will have a contralateral hernia in 13%-26% of cases upon exploration [25,26]. One in five of these patients eventually required repair when followed up for a median of 112 months [27]. Early repair of laparoscopically detected occult contralateral hernia has been effective in preventing progression and complications [20]. Our study identified 31 cases in the TAPP group in which the surgery was initially planned as unilateral but was extended to bilateral during the procedure. When we analyzed these, the mean surgery duration was approximately 61 minutes, which is slightly higher than the overall average for TAPP procedures (p = .035). The mean hospital stay for these patients was 1.065 days, consistent with the overall findings for TAPP (p = .992). The analysis of complications in these cases revealed that the most common complications were cord edema (three cases) and seroma (two cases), consistent with the overall findings for TAPP (p = .208). In our study, it was observed that contralateral hernia repair did not increase the risk of complications.

Given the high incidence of contralateral hernias in patients with symptomatic unilateral hernias, conducting a thorough examination and using ultrasound in suspect cases may be particularly appropriate for surgeries performed using the TEP technique. In our clinic, we perform contralateral repair in the same session for patients who have provided preoperative consent.

Complications

 *Organ and Artery Injury*

The most critical intraoperative complications of inguinal hernia repair are visceral and vascular injuries. Such injuries are rare, typically occurring in less than 1% of cases [9,28]. Many studies note that TAPP procedures have a higher risk of organ injury compared to TEP, primarily due to the transabdominal nature of the procedure, which requires navigation around and manipulation of intra-abdominal organs and brings the risk of IEA injuries, indicating that these are more common in TAPP due to the anatomical exposure required during the procedure [7,15,24,28,29]. In our study, the organ injury rate in TAPP was one case (0.5%), with no injuries reported in TEP. The artery injury rate was also one case (0.5%) in TAPP and none in TEP. The rates of serious complications in our study, such as organ and arterial injuries, are consistent with those reported in the literature.

Seroma Formation and Urinary Retention

Some studies report that TAPP may be associated with a slightly increased risk of seroma due to the more extensive dissection required [20]. In some studies, seroma rates typically range from 5% to 15% for both TEP and TAPP procedures [30]. Seromas, while typically benign, can necessitate additional medical intervention if symptomatic. In our study, the incidence of seroma was greater in the TAPP group (eight cases, 4%) than in the TEP group (six cases, 12%), but the difference was not statistically significant.

Our literature search found urinary retention rates typically varying from 1% to 10%, with TEP often having slightly higher rates than TAPP [7,15]. In our study, the urinary retention rate was 16.0% in TEP and 2.0% in TAPP. Our observed rate of 16.0% in TEP is notably higher than what is generally reported, suggesting a potential area for further investigation. The findings in our data align with the reports in our literature search, indicating that both TEP and TAPP are relatively safe concerning these complications.

Limitations

The limitations of the study include its retrospective design. All operations were performed by two different surgeons. Other limitations were the absence of balloon use in the TEP technique and the difficult learning curve of TEP operations, which could cause a longer operation time. We did not use sutures to close the peritoneum, so using tacks in the TAPP technique may have resulted in shorter operation times. The number of TEP patients was less than the number of TAPP patients. It could affect the learning curve of both techniques. Our follow-up time was one month, so the lack of long-term follow-up may have decreased the recurrence rate. A prospective and single-surgeon design study containing the same quality, a greater number of patients, and a longer follow-up could increase the study’s power.

## Conclusions

This study underscores the importance of selecting the appropriate technique based on the patient’s specific condition, the complexity of the hernia, and the surgeon’s expertise. TAPP offers the benefit of shorter surgery times and better visualization, whereas TEP minimizes the risk of intra-abdominal complications. Both techniques are effective and reduce the recurrence rate, but their success depends largely on the surgeon’s experience and the specific clinical context. Further research is warranted to explore the factors affecting TAPP and TEP and to optimize patient outcomes across both techniques.
